# Burden in Colombia of COVID-19 in Adults and the Associated Clinical Characteristics: A Retrospective Database Analysis

**DOI:** 10.3390/tropicalmed10060146

**Published:** 2025-05-22

**Authors:** Jair Arciniegas, Juan Manuel Reyes, Jhon Bolaños-López, Julia Regazzini Spinardi, Jingyan Yang, Farzaneh Maleki, Farley Johanna Gonzalez, Carlos Jose Bello, Ana Catalina Herrera-Díaz, Omar Escobar, Andrea Rubio, Monica Garcia, Luz Eugenia Pérez Jaramillo, Jorge La Rotta, Moe H. Kyaw, Carlos Fernando Mendoza

**Affiliations:** 1 Pfizer, Bogotá 111211, Cundinamarca, Colombia; 2 Suramericana IPS—Sura, Medellín 050015, Antioquia, Colombiacatalina.herrera.doc.medicina@gmail.com (A.C.H.-D.);; 3 Pfizer, Sao Paulo 04230-040, Brazil; 4 Pfizer, New York, NY 10001, USA; 5 Pfizer, Mexico City 05120, Mexico

**Keywords:** burden of disease, COVID-19, Colombia, incidence, risk factors, adult

## Abstract

Studies on the burden of COVID-19 cases in Colombia have focused on specific populations and short timeframes. A retrospective observational study was conducted on adult patients aged 18 diagnosed with COVID-19 who received inpatient and/or outpatient medical care at a large health maintenance organization, to evaluate the burden of COVID-19 cases in Colombia (from March 2020 to January 2023) and associations with demographic and clinical characteristics. COVID-19 cases were identified with ICD-10 codes and confirmed by a laboratory test. The statistical analysis focused on descriptors of the frequency of events. A multivariate regression model was used to identify factors associated with severe conditions and death. Of the 953,661 cases detected, most cases (~79%) were mild or moderate (handled as outpatients). There were 20.1% (N = 191,260) severe cases and 0.9% (N = 8841) critical cases. Most COVID patients were unvaccinated (94.6%) and had, on average, one comorbidity. Hypertension (19.1%), immunocompromised condition (23.8%), mental health conditions (15%), obesity (10.8%), and cancer (11.2%) were the common prevalent comorbidities. The presence of comorbidity increased the risk of severe or critical COVID-19. COVID-19 cases were associated with the lack of vaccination and comorbidities. Effective vaccination strategies are needed to reduce the burden of COVID-19 in Colombia and, considering budgetary constraints, it is advisable to prioritize the elderly or populations with underlying conditions.

## 1. Introduction

The burden of coronavirus disease (COVID-19) has been vast and multifaceted, affecting countries worldwide with different degrees of severity, leading to unprecedented health crises, economic disruptions, and social challenges [1,2].

COVID-19 manifests itself in many degrees of severity, ranging from no symptoms to a severe illness that may involve respiratory failure, septic shock, and problems with multiple organs [3]. Although most cases of COVID-19 are asymptomatic, mild, or moderate, they run the underlying risk of becoming severe or even life-threatening. Moreover, symptoms can persist and debilitate the patient [4]. According to some studies, around 5–10% of patients with the disease require hospitalization [5]. Those most at risk have a chronic illness or are immunocompromised, pregnant, or over 65 years of age [3,6].

Different resources have been developed in Colombia to mitigate the impact of COVID-19, with appropriate measures coordinated through multiple public policies [7]. For instance, vaccination against COVID-19 was implemented in accordance with a national plan, which began on 17 February 2021. The prioritized populations were soon extended to all adults on July 17 of the same year [8]. However, the disease continues to represent a public health problem, as evidenced by the current Ten-Year Public Health Plan [9]. The constant evolution of the disease remains a concern, as well as the changes in the behavior of the general population regarding awareness, prevention, and vaccination [10,11].

Although different studies have been carried out in the country to understand the characteristics of patients infected with COVID-19, they have each approached the issue with a specific focus. Research has been conducted with short timeframes [12] and on populations in certain cities [13], the elderly [14], patients with specific risk factors [15], and cases involving hospitalization [16,17]. To comprehend the evolution of the disease and make appropriate public policies, it is crucial to analyze the demographic and clinical characteristics of COVID-19 cases over the extended period of the pandemic. Therefore, the current contribution aimed to evaluate the burden of COVID-19 cases in Colombia (from March 2020 to January 2023) and associations with demographic and clinical characteristics by analyzing a large sample of adult databases.

## 2. Materials and Methods

### 2.1. Study Design and Population

A retrospective observational study was carried out on adult patients diagnosed with COVID-19. The sample population had received inpatient and/or outpatient medical care at one of the largest health maintenance organizations (HMOs) in Colombia, which covers 10.08% of the population (~5 million people), according to the Ministry of Health [18]. The data were extracted from an integrated clinical records database from March 2020 to January 2023, identifying adult cases using International Classification of Diseases 10th Revision codes for COVID-19. A diagnosis had to be confirmed with a laboratory test for a case to be considered.

This study used structured databases and was approved by the institutional ethical committee of SURA (Comité de ética y BPC en investigación en salud, CEI-SURA) by Act No. 101 dated 19 July 2023. Informed consent was unnecessary because of the retrospective study design, implying minimal risk and no patient intervention.

The observation window for a COVID-19 case refers to the period from the moment the diagnosis of COVID-19 was confirmed (index date) until the end of follow-up. This period lasted for 45 days after the index date for outpatients or hospitalization.

The COVID-19 cases were categorized by severity according to the World Health Organization (WHO) guidelines and its healthcare resource utilization [19]. Mild cases were managed through outpatient care or by telemedicine or phone consultations. Moderate cases were treated on an outpatient basis or at home with oxygen therapy. Severe cases required hospitalization, and critical cases involved treatment in the intensive care unit.

We defined the high-risk age group as individuals over 60 or 65 years of age, primarily due to the complications generated by a COVID-19 infection [20,21]. The risk factors for severe COVID-19 were herein identified in accordance with the guidelines provided by the Centers for Disease Control and Prevention (CDC) [21]. In addition to the aforementioned age factor, the risk factors included a history of cancer, chronic kidney disease (CKD), chronic lung disease, dementia or other neurological condition, type 1 or type 2 diabetes, Down syndrome, cardiovascular disease, Human Immunodeficiency Virus (HIV) infection or Acquired Immunodeficiency Syndrome (AIDS), hypertension, an immune deficiency or immunocompromised condition, liver disease, obesity, sickle-cell disease, thalassemia, current or past smoking, and a solid organ or blood transplant [21]. Reinfection was considered if a COVID infection took place 45 days or more after the previous infection [22,23,24].

Vaccination status is classified as follows:

- unvaccinated: without any COVID-19 vaccination record
- partially vaccinated: a single dose of the messenger ribonucleic acid (mRNA) or adenoviral vector vaccines
- fully vaccinated: two doses of the mRNA or adenoviral vector vaccines, or a single dose of the Johnson & Johnson vaccine
- fully vaccinated + booster: fully vaccinated and some additional COVID-19 vaccine dose

Finally, patient death is considered to be related to COVID-19 if it occurred within the previously defined timeframe for the disease.

### 2.2. Statistical Analysis

Descriptive statistics were generated to analyze demographic and clinical variables. We used percentages and frequencies to describe dichotomic, ordinal, and nominal variables, while mean and standard deviation (SD) were calculated for quantitative variables.

To comprehend the evolution of the burden of COVID-19 during different timeframes, indicators such as the monthly and annual cumulative frequency, the crude mortality rate, and the case fatality rate were calculated from the frequency of occurrence of events. The incidence rate was calculated as (new cases/population) × 100,000. The mortality rate was ascertained using a similar approach.

To explore the changes in the burden of disease associated with the introduction of vaccination strategies at the national level, a subgroup analysis was performed using the first infection cases. The test of equal or given proportions was employed to assess statistically relevant changes within groups.

Two multivariate logistic regression models were used. In one, the dependent variable was a severe or critical COVID-19 case, and in the other, a deceased patient. Associations were assessed based on patient characteristics. A statistical analysis was performed using R statistical language (v. 4.3.1) [25], with significance set at 5%.

## 3. Results

### 3.1. Demographic and Clinical Characteristics

A total of 953,661 adult cases qualified for inclusion in the study, corresponding to 894,326 patients. The mean age was 42.3 years (±15), with 18–49-year-olds comprising most cases (71.2%, n = 678,559), followed by 50–64-year-olds (20.6%), and then the elderly (65 years of age or older; 8.3%). Around 56% of the sample was female, 57.1% lived in Antioquia, almost all lived in urban areas (99.4%), and of the cases that reported data, the most reported being of mixed race (24.4%) (Table 1). The majority (61.7%) reported earnings under two times the minimum wage (In 2022, this corresponded to subjects with a monthly income below 470 United States Dollars (USD)).

The mean weight was 68.5 kg (±22.2) and the average height was 151.1 cm (±45.5). Only 0.5% of the cases involved pregnancy. Most of the sample had a mild or moderate condition (79%) handled in outpatient care, while 20.1% of the cases were severe, and 0.9% critical. Reinfected cases constituted 6.2% of the sample. The average number of comorbidities was 1.0 (±1.4), and the most frequent comorbidities were an immunocompromised condition (23.8%, n = 226,623), hypertension (19.1%), a mental health condition (15%), cancer (11.2%), and obesity (10.8%). Medication was taken chronically by 45.5% of the patients. A risk factor for severe COVID-19 was found in about 9.1% of all COVID-19 cases (Table 1).

Most cases (94.6%, n = 901,966) were unvaccinated, and only a small proportion (3.4%, n = 32,823) were fully vaccinated. Vaccinated cases (partial, full, or full plus booster) had a higher average age and number of comorbidities and tended to be concentrated among females. It was more common for subjects from higher-income households to be vaccinated, and for vaccinated patients to have milder conditions than those unvaccinated (Table 1).

### 3.2. Frequency of Cases by Year and Age Group

**Figure 1:**
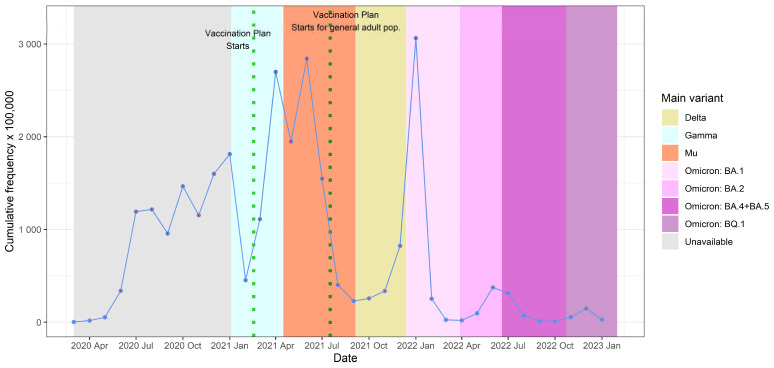
Monthly cumulative frequency of confirmed cases and variant predominance in Colombia, March 2020 to January 2023. Note 1: Variant predominance was based on data from the National Health Institute (INS, by its acronym in Spanish) [26] and Holdcroft et al. [27]. Note 2: The vaccination plan began on 17 February 2021, with prioritized populations such as healthcare workers; later, on July 17 of the same year, it was extended to all adults.

COVID-19 cases were more frequent in 2021 (54.9%), followed by 26.8% in 2020. Among the different variants over time in Colombia [26,27], the Mu variant dominance period was associated with the highest percentage of cases (21%) (Figure 1).

The monthly cumulative frequency of COVID-19 cases reveals a multimodal behavior with several peaks. The Colombian vaccination plan started [8] on 17 February 2021, and was extended to all adults on 17 July 2021. The peaks were successively higher as time passed until reaching the greatest number of cases in January 2022, after which time the number of cases per month diminished. The first peak (in April 2021, the predominance of the Gamma variant) was constituted by 2698.7 cases per 100,000 members of the HMO (confidence interval [CI] 95%; 2681.4–2716.1). The second peak (in June 2021, predominance of the Mu variant) consisted of 2841.5 cases per 100,000 members (CI 95%; 2823.8–2859.2). The third peak (in January 2022, the predominance of the Omicron BA.1 variant) comprised 3063.2 cases per 100,000 members (CI 95%; 3045.4–3081.0). This general pattern is similar to that found in other reports, which also describe the highest peak in January 2022 [28,29].

**Figure 2:**
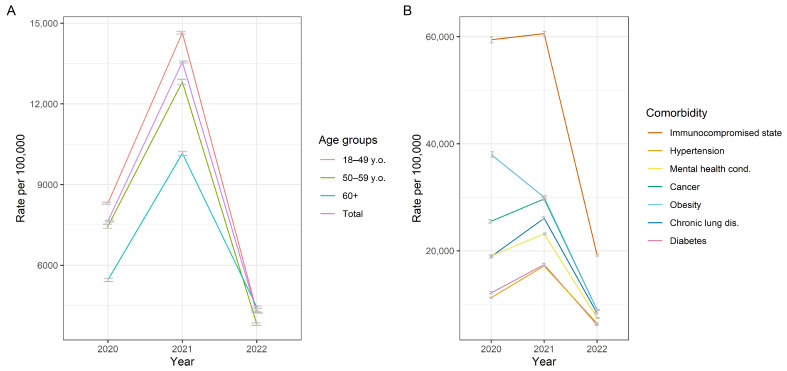
Annual frequency of COVID-19 cases from 2020 to 2022 by age groups (**A**) and main comorbidities (**B**).

The annual pattern of cases from 2020–2022 displays an inverted U-shape, with 7640 cases per 100,000 members of the HMO (CI 95%; 7610–7671) in 2020, rising to 13,559 (CI 95%; 13,520–13,598) in 2021, and falling to 4237 (CI 95%; 4217–4257) in 2022. The greatest risk of developing COVID-19 corresponded to middle-aged adults (18–49 years of age) (Figure 2A). The presence of comorbidities led to a 1.25- to 7.78-fold increase in the risk of infection. For example, patients with an immunocompromised condition showed an annual frequency of 59,421 cases per 100,000 members in 2020 (CI 95%; 58,835–60,011), 60,557 in 2021 (CI 95%; 60,119–60,998), and 19,214 in 2022 (CI 95%; 19,023–19,405), which indicates a 7.78-, 4.47-, and 4.53-fold greater risk of infection, respectively, compared to the overall sample (Figure 2B).

### 3.3. Severity and Vaccination Status

A lower proportion of critical and severe cases was observed for the vaccinated versus unvaccinated patients. Critical cases constituted 0.9% of the total number of unvaccinated subjects in the sample (8846/901,966) and 0.5% of those fully vaccinated (151/32,823). This pattern is even more prominent for severe cases, which made up 20.4% of the total number of unvaccinated subjects (184,014/901,966) and 9.5% of those fully vaccinated (3116/32,823). Hence, the proportion of mild cases was slightly higher among the vaccinated than unvaccinated patients, being 88.1% (28,907/32,823) for the former and 78.0% (703,237/901,966) for the latter (Appendix A).

**Figure 3:**
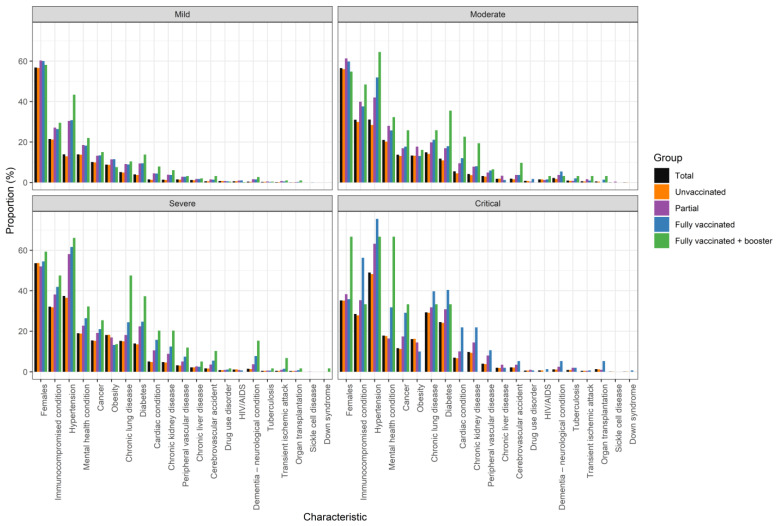
COVID-19 severity in relation to vaccination status, gender, and the presence of comorbidities.

With a greater age, the risk of the severity of the disease increased. Indeed, the mean age for the mild cases was 40.2 (±13.8) years, and for the critical cases, it was 58.8 (±14.6) years (Appendix A). Additionally, individuals with certain comorbidities represented a larger proportion of total critical cases than their proportion of total mild cases. For example, individuals with an immunocompromised condition were responsible for 21.5% of mild cases and 28.5% of critical cases. Moreover, individuals with hypertension accounted for 13.9% of mild cases and 49.0% of critical cases, and individuals with mental health conditions were responsible for 13.9% of mild cases and 17.8% of critical cases (Figure 3).

These patterns are exacerbated by variations in vaccination status. For instance, the mean age for mild cases was 39.6 ± 13.3 years for unvaccinated patients and 58.3 ± 14.4 years for fully vaccinated patients. Meanwhile, for critical cases, it was 50.1 ± 16.7 years for unvaccinated patients and 73.9 ± 12.3 years for fully vaccinated patients (Appendix A).

The logistic regression model confirmed that a greater age and/or weight, male sex, a higher number of comorbidities, and especially reinfection (compared to first infections, ceteris paribus, reinfection cases had a 2.9-fold greater risk of being severe or critical COVID-19) have a positive and statistically significant association with the probability (odds ratio, OR) of developing severe or critical COVID-19. Conversely, full vaccination status and a higher income tend to decrease the same probability (OR) (Table 2).

On the other hand, there was a difference in the proportion of severe and critical cases before and after the start of the vaccination plan for all adults (17 July 2021). Severe cases constituted 24.6% of the total cases before vaccination began and 8.3% of the total cases afterwards. Critical cases represented 1.1% (7762/685,840) before vaccination began and 0.4% (1079/267,821) afterwards. Both differences were statistically significant according to the test of equal or given proportions. The opposite tendency can be appreciated when comparing the proportion of these cases between 2022 (Appendix B provides annual figures for 2022 on the distribution of death cases by care setting and age, among other characteristics) and 2023 (taking into account that in the latter year, the data is only available for January). In 2022, the severe cases were at 6.6% of the total cases (11,396/173,770), and at 11.0% (127/1156) in January 2023. Similarly, in 2022, the critical cases were at 0.3% (455/173,770), and at 0.8% (9/1156) in January 2023. Both differences were statistically significant.

### 3.4. Trends by Risk Factor

Considering the first-infection cases, 894,876 were analyzed. Individuals 65 years of age or older constituted 8.2% (n = 55,287) of all cases before the vaccination plan began, and 12.6% (n = 28,179) afterwards. They had a lower proportion of cases with comorbidity subsequent to (versus prior to) the implementation of the plan (83.6% vs. 84.8%). This same pattern held for certain comorbidities such as hypertension (67.8% vs. 68.5%), obesity (8.2% vs. 9.5%), chronic lung disease (21.1% vs. 23.1%), and diabetes (25.6% vs. 28.3%). For other comorbidities, however, the reverse pattern occurred, with a higher proportion found after the vaccination plan began. Examples include a mental health condition (24.5% vs. 21.9%), cancer (19.6% vs. 18.7%), and dementia (7.6% vs. 5.2%). No significant differences were detected in this regard for some comorbidities, such as chronic kidney disease, chronic liver disease, an immunocompromised condition, HIV, tuberculosis, and sickle cell disease (Table 3).

### 3.5. Mortality and Case Fatality Rate

**Figure 4:**
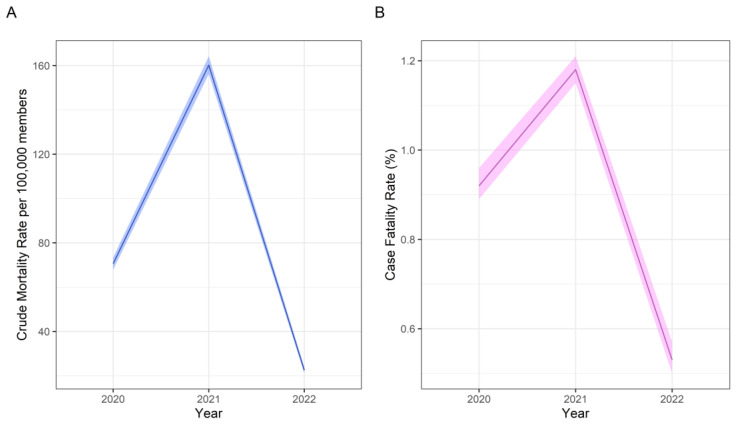
Annual crude mortality rate (**A**) and case fatality rate (**B**).

Analogous to the trend observed with case frequency, the annual pattern of the crude mortality rate showed an inverted U-shape. It was 70.6 per 100,000 members of the HMO (CI 95%; 67.81–73.43) in 2020, 160.2 (CI 95%; 156.19–164.22) in 2021, and 22.5 (CI 95%; 21.07–23.97) in 2022. Likewise, the annual case fatality rate was 0.92% (CI 95%; 0.89–0.96%) in 2020, 1.18% (CI 95%; 1.15–1.21%) in 2021, and 0.53% (CI 95%; 0.50–0.57%) in 2022 (Figure 4).

On the other hand, logistic regression revealed the association of an increased probability of death (measured by the odds ratio, OR) with greater age or weight, a higher number of comorbidities, and a more severe condition (compared to mild cases, critical cases have a higher probability (measured by OR) of death). Moreover, there were more deaths among males than females, and proportionally more (versus total cases) in January 2023 compared to the annual figures for 2022 (2023 = 53.6% males vs. 2022 = 52.7% males).

Contrarily, some clinical characteristics have an inverse relationship, decreasing the risk of death, such as a full vaccination status, a higher income, and reinfection (Table 2).

## 4. Discussion

The present study evaluated the burden of COVID-19 cases of adults in a large HMO in Colombia between March 2020 and January 2023. Almost 1 million cases were found during this timeframe, most of them were mild or moderate (handled as outpatients), with a low household income, and around 42 years old. The burden of severe and critical cases tended to be borne by males, aged 49 years and older, unvaccinated, and with comorbidities.

The monthly burden of disease reached its third and highest peak in January 2022, with over 2600 cases per 100,000 members of the HMO. During the last 11 months of observation, the incidence did not exceed 400 cases per 100,000 members. The frequency and mortality rate varied substantially in accordance with the different waves of COVID variants.

The current analysis provides insights into the clinical and demographic characteristics of COVID-19 cases during the first 35 months since its appearance in Colombia. Over 90% of the sample comprised urban residents and unvaccinated individuals. There were slightly more cases of females than males, except in relation to critical cases. The group of 18–49-year-olds constituted the majority of cases, but the elderly had a greater risk of a severe or critical condition. Moreover, the existence of comorbidity increased the risk of severe or critical COVID-19. The general findings about the demographical and clinical characteristics are consistent with publications in the literature, particularly with those who reported greater severity of COVID-19 associated with obesity and being male and/or elderly [4,30,31,32].

Due to the priorities of the Colombian vaccination plan, most vaccinated cases were older adults. Thus, a higher number of comorbidities was found among vaccinated than unvaccinated patients. The plan was divided into stages, with priority given as follows: (i) the population over 80 years of age and healthcare workers; (ii) police, military, and the population from 60 to 79 years old; (iii) educational workers and teachers, caregivers, and the population of 16–59 years of age with a risk factor (e.g., hypertension, diabetes, kidney failure, HIV, cancer, tuberculosis, asthma, and obesity); (iv) the incarcerated population, homeless people, and high-risk professions; and finally, (v) the population 16 years old and over and groups not mentioned in the previous stages, starting with the population from 50 to 59 years of age [8].

Similar to observations reported in other cities and regions, the results suggest that the Colombian vaccination plan is related to a decrease in the absolute frequency of cases across all groups and a reduction in the severity of the cases [14,33] However, certain groups at risk did not receive special protective measures, particularly in the group with mental and neurological disorders, such as dementia or a mental health condition. On the other hand, some authors argue that immunocompromised cases are not all at high risk of COVID-19, but rather that there are subgroups within this group that differ in their prognosis and risk [34].

Despite the national vaccination plan not prioritizing income or socioeconomic status, and most cases in the study sample belonging to households with an income under two times the minimum wage, vaccinated cases tended to be concentrated in higher-income households, and it can be argued that this behavior represents social inequality. According to the data, a full vaccination reduces the probability of death and a severe or critical condition. Also, promoting a protective influence against death from COVID-19 was reinfection, although a greater probability existed of developing a severe or critical condition. A worrisome result is the increase in the proportion of severe and critical cases and of the OR of death in January of 2023 compared to the annual data in 2022 and 2020, respectively.

At the time of writing this manuscript, virtually no published studies collecting data beyond 2023 have been found in Latin America, leaving as the main reference dashboards with highly aggregated information, which for Colombia indicated a recent peak in August 2024 that did not exceed 4000 cases [35]. In this context, as Ulrichs et al. [36] mentioned, some variations of the COVID-19 epidemiology and burden are expected to occur, those being an increase in immunized cases, a decrease in disease severity, a reduction in detection, and further co-occurrence with other respiratory diseases. This follow-up is required in future studies.

In view of the above findings, the competent authorities and the health sector in general must not let down their guard in at least four aspects. Firstly, it is understandable that disease prevention messages have diminished compared to the period of the health emergency [37], but disease awareness must continue to be present. Secondly, detection and reporting of confirmed cases of COVID-19 should not be neglected. The broad screening guidelines implemented during the emergency have been relaxed, as shown by the daily test per million of 1938 in June 2021 and 357 in June 2022 [38,39,40]. Thirdly, COVID-19 vaccination coverage should be sustained and expanded, considering the problematic behavior of the cases in January 2023. Fourthly, vaccination and prevention campaigns need to be accompanied by strategies to address the spread of misinformation to facilitate evidence-based policy decisions [41], such as partnerships with fact-checking organizations. Azevedo et al. [42] argued that in Brazil, these agencies played an important role in shaping public trust and behavior.

The current contribution in Colombia used a large integrated clinical records database for a diverse sample involving many different healthcare providers and settings, providing a comprehensive landscape of the burden of the disease. Unlike most studies that focus on a specific setting, age group, or limited timeframe, this research provides a broader perspective. Moreover, the study had the ability to split the analyses by different subgroups and time spans, allowing an in-depth characterization of the demographic and clinical characteristics of COVID-19 cases in the country. Nevertheless, some limitations exist, such as the incompleteness of the data on the race variable (missing in over 60% of the cases). This variable has often been key to explaining disparities in COVID-19 outcomes [43,44]. Studies in Latin-American countries have indicated that mixed and black race persons tend to have increased mortality risks [45]. On a similar note, over 60% of cases concentrated in Antioquia and Bogotá are characterized by their location in the Andean mountains, high population density, and leading economic development in the country. This may reduce the comparability of the results to other regions of the country. Moreover, there may have been under-reporting or misclassification of comorbidities. Furthermore, in-depth clinical information was not provided (e.g., symptoms, laboratory test results, and the variant per case), nor was information on some relevant outpatient behavior, such as the use of over-the-counter drugs. Finally, data was not collected on asymptomatic cases. The present study can only demonstrate associations and not causality between variables, and its results cannot be generalized to the entire Colombian population.

In conclusion, COVID-19 cases in Colombia showed peaks in April and June of 2021 and January 2022, each successively higher, followed by a decline in the number of cases. Severe and critical cases were associated with a lack of vaccination, and/or weight, reinfection, and comorbidities (especially hypertension, an immunocompromised condition, a mental health condition, obesity, and cancer). Effective vaccination strategies are needed to reduce the burden of COVID-19 in Colombia, and, considering budgetary constraints, it is advisable to prioritize the elderly or populations with underlying conditions.

## Figures and Tables

**Table 1:** Demographic and clinical characteristics of COVID-19 positive cases in a large HMO (from March 2020 to January 2023).

	Total, n (%)	Unvaccinated, n (%)	Partial, n (%)	Fully Vaccinated ^♣^, n (%)	Fully + Booster ^♣^
	953,661 (100)	901,966 (94.6)	17,973 (1.9)	32,823 (3.4)	899 (0.1)
Demographic data					
Age, mean (SD)	42.3 (15)	41.8 (14.6)	53.1 (18.1)	51.9 (17.6)	61.3 (17.4)
Age groups, n (%)					
18 to 49 years	678,559 (71.2)	654,137 (72.5)	7853 (43.7)	16,327 (49.7)	242 (26.9)
50 to 64 years	196,367 (20.6)	182,395 (20.2)	5159 (28.7)	8589 (26.2)	224 (24.9)
Over 65 years	78,735 (8.3)	65,434 (7.3)	4961 (27.6)	7907 (24.1)	433 (48.2)
Female, n (%)	533,895 (56)	503,428 (55.8)	10,463 (58.2)	19,482 (59.4)	522 (58.1)
Area of residence, n (%)					
Urban	947,945 (99.4)	896,424 (99.4)	17,907 (99.6)	32,716 (99.7)	898 (99.9)
Rural	5442 (0.6)	5272 (0.6)	66 (0.4)	103 (0.3)	1 (0.1)
Unknown	274 (0)	270 (0)	-	4 (0)	-
Region of residence, n (%)					
Antioquia	544,911 (57.1)	513,348 (56.9)	10,016 (55.7)	21,032 (64.1)	515 (57.3)
Atlántico	102,152 (10.7)	95,115 (10.5)	2590 (14.4)	4397 (13.4)	50 (5.6)
Bogotá, Capital City	104,404 (10.9)	101,739 (11.3)	1179 (6.6)	1439 (4.4)	47 (5.2)
Caldas	24,567 (2.6)	22,978 (2.5)	1075 (6)	511 (1.6)	3 (0.3)
Santander	26,153 (2.7)	25,810 (2.9)	289 (1.6)	54 (0.2)	-
Valle del Cauca	88,430 (9.3)	80,653 (8.9)	2478 (13.8)	5021 (15.3)	278 (30.9)
Other	63,044 (6.6)	62,323 (6.9)	346 (1.9)	369 (1.1)	6 (0.7)
Race, n (%)					
Afro-Colombian	7000 (0.7)	6479 (0.7)	172 (1)	343 (1)	6 (0.7)
Mulato	1070 (0.1)	1016 (0.1)	25 (0.1)	28 (0.1)	1 (0.1)
Zambo	437 (0)	408 (0)	10 (0.1)	18 (0.1)	1 (0.1)
Indigenous	148 (0)	134 (0)	9 (0.1)	5 (0)	-
White-Hispanic	68,764 (7.2)	63,218 (7)	1925 (10.7)	3505 (10.7)	116 (12.9)
Mixed	232,454 (24.4)	215,921 (23.9)	5709 (31.8)	10,558 (32.2)	266 (29.6)
Unknown	643,788 (67.5)	614,790 (68.2)	10,123 (56.3)	18,366 (56)	509 (56.6)
Household income, n (%)					
<2 times the minimum wage	588,063 (61.7)	560,839 (62.2)	10,090 (56.1)	16,728 (51)	406 (45.2)
2–5 times the minimum wage	221,304 (23.2)	207,781 (23)	4487 (25)	8781 (26.8)	255 (28.4)
>5 times the minimum wage	88,900 (9.3)	79,413 (8.8)	2755 (15.3)	6504 (19.8)	228 (25.4)
Unknown	55,394 (5.8)	53,933 (6)	641 (3.6)	810 (2.5)	10 (1.1)
Clinical data					
Weight, kg (SD)	69 (22.2)	69 (22.2)	69 (21.3)	68 (22.3)	66 (23.7)
Height, cm (SD)	151 (45.5)	151 (45.2)	150 (45.3)	146 (51.7)	138 (59.6)
Pregnant, n (%)	5235 (0.5)	4783 (0.5)	152 (0.8)	290 (0.9)	10 (1.1)
Number of comorbidities, mean (SD)	1 (1.4)	1 (1.4)	2 (1.7)	2 (1.7)	2 (1.9)
Comorbidities, n (%)					
Immunocompromised condition	226,623 (23.8)	211,700 (23.5)	5370 (29.9)	9271 (28.2)	282 (31.4)
Hypertension	181,883 (19.1)	163,501 (18.1)	6692 (37.2)	11,279 (34.4)	411 (45.7)
Mental health condition	143,341 (15)	133,315 (14.8)	3513 (19.5)	6305 (19.2)	208 (23.1)
Cancer	106,730 (11.2)	99,272 (11)	2625 (14.6)	4688 (14.3)	145 (16.1)
Obesity	102,941 (10.8)	96,752 (10.7)	2292 (12.8)	3823 (11.6)	74 (8.2)
Chronic lung disease	70,959 (7.4)	65,250 (7.2)	2072 (11.5)	3516 (10.7)	121 (13.5)
Diabetes	59,707 (6.3)	53,587 (5.9)	2281 (12.7)	3694 (11.3)	145 (16.1)
Cardiac disorder	21,792 (2.3)	18,754 (2.1)	1071 (6)	1884 (5.7)	83 (9.2)
Chronic kidney disease	20,713 (2.2)	18,193 (2)	916 (5.1)	1537 (4.7)	67 (7.5)
Peripheral vascular disease	17,741 (1.9)	15,989 (1.8)	608 (3.4)	1109 (3.4)	35 (3.9)
Chronic liver disease	13,188 (1.4)	12,235 (1.4)	353 (2)	581 (1.8)	19 (2.1)
Cerebrovascular accident	8166 (0.9)	7169 (0.8)	361 (2)	601 (1.8)	35 (3.9)
Drug use disorder	6964 (0.7)	6614 (0.7)	137 (0.8)	209 (0.6)	4 (0.4)
HIV/AIDS	6720 (0.7)	6235 (0.7)	161 (0.9)	321 (1)	3 (0.3)
Dementia—neurological condition	6249 (0.7)	5118 (0.6)	376 (2.1)	723 (2.2)	32 (3.6)
Tuberculosis	3143 (0.3)	2922 (0.3)	94 (0.5)	122 (0.4)	5 (0.6)
Transient ischemic attack	2745 (0.3)	2405 (0.3)	134 (0.7)	193 (0.6)	13 (1.4)
Organ transplantation	1913 (0.2)	1712 (0.2)	52 (0.3)	139 (0.4)	10 (1.1)
Sickle cell disease	451 (0)	425 (0)	10 (0.1)	16 (0)	-
Down syndrome	132 (0)	119 (0)	4 (0)	8 (0)	1 (0.1)
Risk factors for severe COVID-19, n (%)					
Age and a comorbidity	86,990 (9.1)	72,812 (8.1)	5284 (29.4)	8427 (25.7)	467 (51.9)
Age only	14,994 (1.6)	12,591 (1.4)	847 (4.7)	1456 (4.4)	100 (11.1)
Severity, n (%)					
Mild	746,408 (78.3)	703,237 (78)	13,458 (74.9)	28,907 (88.1)	806 (89.7)
Moderate	7152 (0.7)	6229 (0.7)	243 (1.4)	649 (2)	31 (3.4)
Severe	191,260 (20.1)	184,014 (20.4)	4071 (22.7)	3116 (9.5)	59 (6.6)
Critical	8841 (0.9)	8486 (0.9)	201 (1.1)	151 (0.5)	3 (0.3)
Reinfected, n (%)	58,785 (6.2)	57,143 (6.3)	648 (3.6)	975 (3)	19 (2.1)
Number of reinfections, n (%)					
1	52,099 (5.5)	50,622 (5.6)	578 (3.2)	881 (2.7)	18 (2)
2	2559 (0.3)	2491 (0.3)	39 (0.2)	29 (0.1)	-
3	125 (0)	123 (0)	2 (0)	-	-
4	4 (0)	4 (0)	-	-	-
Chronic Medication Usage, n (%)					
1 Medication	327,463 (34.3)	311,172 (34.5)	5884 (32.7)	10,217 (31.1)	190 (21.1)
2 Medications	63,887 (6.7)	57,952 (6.4)	2198 (12.2)	3588 (10.9)	149 (16.6)
3 Medications	31,593 (3.3)	27,702 (3.1)	1470 (8.2)	2338 (7.1)	83 (9.2)
4 Medications	10,430 (1.1)	8981 (1)	570 (3.2)	841 (2.6)	38 (4.2)
5 Medications	434 (0)	351 (0)	29 (0.2)	53 (0.2)	1 (0.1)
None	519,854 (54.5)	495,808 (55)	7822 (43.5)	15,786 (48.1)	438 (48.7)

^♣^ Note: Fully vaccinated: two doses of the mRNA or adenoviral vector vaccines, or a single dose of the Johnson & Johnson vaccine. Fully vaccinated + booster: fully vaccinated and some additional COVID-19 vaccine dose.

**Table 2:** Logistic regression model results using a severe or critical condition as the dependent variable (Model 1) and using death as the dependent variable (Model 2).

	Model 1: Severity ^♣^	Model 2: Death ^♣^
	OR [CI 95%] (*p*-value)	OR [CI 95%] (*p*-value)
(Intercept)	0.015 ***	0.000 ***
	[0.015, 0.016] (<0.001)	[0.000, 0.000] (<0.001)
Age	1.041 ***	1.091 ***
	[1.040, 1.041] (<0.001)	[1.089, 1.093] (<0.001)
Male	1.043 ***	1.775 ***
	[1.031, 1.055] (<0.001)	[1.685, 1.870] (<0.001)
Weight (kg)	1.017 ***	0.989 ***
	[1.016, 1.017] (<0.001)	[0.988, 0.990] (<0.001)
Height (cm)	1.000 *	
	[1.000, 1.000] (0.012)	
Between two and five legal minimum wages	0.954 ***	0.859 ***
	[0.942, 0.967] (<0.001)	[0.804, 0.916] (<0.001)
Income: Missing	0.908 ***	0.721 ***
	[0.886, 0.929] (<0.001)	[0.635, 0.816] (<0.001)
More than five legal minimum wages	0.713 ***	0.716 ***
	[0.698, 0.729] (<0.001)	[0.642, 0.797] (<0.001)
Fully vaccinated	0.471 ***	0.665 ***
	[0.452, 0.491] (<0.001)	[0.585, 0.753] (<0.001)
Fully vaccinated + booster	0.354 ***	0.656
	[0.265, 0.464] (<0.001)	[0.331, 1.164] (0.185)
Partially vaccinated	1.036+	0.826 **
	[0.995, 1.078] (0.090)	[0.723, 0.941] (0.005)
Reinfected	2.938 ***	0.651 ***
	[2.868, 3.008] (<0.001)	[0.550, 0.765] (<0.001)
Number of commorbidities	1.304 ***	1.140 ***
	[1.298, 1.309] (<0.001)	[1.113, 1.168] (<0.001)
Year: 2021	0.661 ***	1.509 ***
	[0.654, 0.669] (<0.001)	[1.421, 1.605] (<0.001)
Year: 2022	0.103 ***	0.967
	[0.101, 0.106] (<0.001)	[0.877, 1.067] (0.510)
Year: 2023	0.119 ***	1.952 **
	[0.098, 0.144] (<0.001)	[1.238, 2.951] (0.002)
Critical		851.564 ***
		[766.605, 946.666] (<0.001)
Moderate		3.412 ***
		[2.389, 4.770] (<0.001)
Severe		3.473 ***
		[3.158, 3.821] (<0.001)
Number of commorbidities × Critical		0.512 ***
		[0.494, 0.531] (<0.001)
Number of commorbidities × Moderate		0.870 **
		[0.787, 0.960] (0.006)
Number of commorbidities × Severe		0.847 ***
		[0.823, 0.873] (<0.001)
Num. Obs.	953,408	953,414
AIC	820,221	54,654
BIC	820,409	54,901
Log. Lik.	−410,094	−27,306
F	7778	1571
RMSE	0.37	0.08

^♣^ Note: + *p* < 0.1, * *p* < 0.05, ** *p* < 0.01, *** *p* < 0.001.

**Table 3:** Proportion of cases for each comorbidity prior to and subsequent to the implementation of the vaccination plan for all adults, categorized by age group.

		Previous to Vaccination Plan		Subsequent to Vaccination Plan ^♣^	
		<65, n (%)	≥65, n (%)	<65, n (%)	≥65, n (%)
		615,608 (100)	55,287 (100)	195,802 (100)	28,179 (100)
Without Comorbidities		328,983 (53.4)	8411 (15.2)	104,499 (53.4)	4630 (16.4) *
Comorbidities					
	Total	286,625 (46.6)	46,876 (84.8)	91,303 (46.6) *	23,549 (83.6) *
	Immunocompromised state	134,145 (21.8)	21,032 (38)	43,291 (22.1) *	10,898 (38.7)
	Hypertension	85,169 (13.8)	37,888 (68.5)	28,227 (14.4) *	19,108 (67.8) *
	Mental health conditions	85,303 (13.9)	12,101 (21.9)	28,162 (14.4) *	6900 (24.5) *
	Cancer	62,758 (10.2)	10,355 (18.7)	20,364 (10.4) *	5536 (19.6) *
	Obesity	66,772 (10.8)	5262 (9.5)	20,482 (10.5) *	2322 (8.2) *
	Chronic lung disease	35,078 (5.7)	12,746 (23.1)	11,417 (5.8) *	5939 (21.1) *
	Diabetes	25,472 (4.1)	15,620 (28.3)	7790 (4) *	7225 (25.6) *
	Cardiac conditions	7240 (1.2)	6926 (12.5)	2480 (1.3) *	3800 (13.5) *
	Chronic kidney disease	7264 (1.2)	6514 (11.8)	2365 (1.2)	3351 (11.9)
	Peripheral vascular disease	8000 (1.3)	3716 (6.7)	2759 (1.4) *	2080 (7.4) *
	Chronic liver disease	7659 (1.2)	1200 (2.2)	2588 (1.3) *	609 (2.2)
	Cerebrovascular accident	2893 (0.5)	2329 (4.2)	910 (0.5)	1462 (5.2) *
	HIV/AIDS	4329 (0.7)	163 (0.3)	1493 (0.8) *	81 (0.3)
	Dementia—Neurological conditions	659 (0.1)	2892 (5.2)	239 (0.1)	2142 (7.6) *
	Tuberculosis	1855 (0.3)	272 (0.5)	615 (0.3)	159 (0.6)
	Transient ischemic attack	1068 (0.2)	700 (1.3)	376 (0.2)	427 (1.5) *
	Organ transplantation	911 (0.1)	249 (0.5)	418 (0.2) *	164 (0.6) *
	Sickle cell disease	270 (0)	20 (0)	120 (0.1) *	8 (0)
	Down syndrome	92 (0)	(0)	35 (0)	2 (0)

^♣^ Note: When the result of the test of equal or given proportions indicated a statistically significant difference between the <65 and ≥65 group, an asterisk (*, *p* < 0.05) was used in the second column (“subsequent to vaccination plan”).

## Data Availability

The original contributions presented in this study are included in the article. Further inquiries can be directed to the corresponding author.

## Appendix A. COVID-19 Severity in Relation to Vaccination Status, Age, Gender, and the Presence of Comorbidities

			**Total**	**Unvaccinated**	**Partial**	**Fully Vaccinated**	**Fully + Booster**
Mild, n (%)			746,408 (100)	703,237 (94.2)	13,458 (1.8)	28,907 (3.9)	806 (0.1)
	Age, mean (SD)		40.2 (±13.8)	39.6 (±13.3)	50.1 (±17.8)	50.1 (±16.7)	60.4 (±17.1)
	Female, n (%)		424,148 (56.8)	398,218 (56.6)	8121 (60.3)	17,341 (60)	468 (58.1)
	Comorbidities, n (%)	Immunocompromised condition	160,426 (21.5)	148,899 (21.2)	3652 (27.1)	7637 (26.4)	238 (29.5)
		Hypertension	103,813 (13.9)	90,457 (12.9)	4096 (30.4)	8910 (30.8)	350 (43.4)
		Mental health condition	103,943 (13.9)	96,010 (13.7)	2488 (18.5)	5268 (18.2)	177 (22)
		Cancer	75,321 (10.1)	69,552 (9.9)	1773 (13.2)	3875 (13.4)	121 (15)
		Obesity	65,886 (8.8)	60,980 (8.7)	1532 (11.4)	3313 (11.5)	61 (7.6)
		Chronic lung disease	38,191 (5.1)	34,325 (4.9)	1222 (9.1)	2560 (8.9)	84 (10.4)
		Diabetes	30,074 (4)	25,947 (3.7)	1268 (9.4)	2748 (9.5)	111 (13.8)
		Cardiac condition	11,063 (1.5)	9113 (1.3)	602 (4.5)	1284 (4.4)	64 (7.9)
		Chronic kidney disease	10,306 (1.4)	8683 (1.2)	508 (3.8)	1066 (3.7)	49 (6.1)
		Peripheral vascular disease	11,020 (1.5)	9800 (1.4)	373 (2.8)	821 (2.8)	26 (3.2)
		Chronic liver disease	8599 (1.2)	7862 (1.1)	230 (1.7)	491 (1.7)	16 (2)
		Cerebrovascular accident	4492 (0.6)	3871 (0.6)	198 (1.5)	397 (1.4)	26 (3.2)
		Drug use disorder	5333 (0.7)	5067 (0.7)	99 (0.7)	164 (0.6)	3 (0.4)
		HIV/AIDS	4353 (0.6)	3944 (0.6)	121 (0.9)	286 (1)	2 (0.2)
		Dementia—neurological condition	3121 (0.4)	2452 (0.3)	211 (1.6)	436 (1.5)	22 (2.7)
		Tuberculosis	2100 (0.3)	1948 (0.3)	63 (0.5)	86 (0.3)	3 (0.4)
		Transient ischemic attack	1647 (0.2)	1412 (0.2)	88 (0.7)	139 (0.5)	8 (1)
		Organ transplantation	980 (0.1)	845 (0.1)	31 (0.2)	96 (0.3)	8 (1)
		Sickle cell disease	345 (0)	325 (0)	5 (0)	15 (0.1)	-
		Down syndrome	85 (0)	75 (0)	3 (0)	7 (0)	-
Moderate, n (%)			7152 (100)	6229 (87.1)	243 (3.4)	649 (9.1)	31 (0.4)
	Age, mean (SD)		47.7 (17.6)	46.2 (16.9)	55. (20.2)	57.7 (19.3)	62.9 (18.7)
	Female, n (%)		4044 (56.5)	3490 (56)	149 (61.3)	388 (59.8)	17 (54.8)
	Comorbidities, n (%)	Immunocompromised condition	2220 (31)	1864 (29.9)	97 (39.9)	244 (37.6)	15 (48.4)
		Hypertension	2226 (31.1)	1767 (28.4)	102 (42)	337 (51.9)	20 (64.5)
		Mental health condition	1504 (21)	1259 (20.2)	68 (28)	167 (25.7)	10 (32.3)
		Cancer	979 (13.7)	815 (13.1)	41 (16.9)	115 (17.7)	8 (25.8)
		Obesity	953 (13.3)	820 (13.2)	43 (17.7)	85 (13.1)	5 (16.1)
		Chronic lung disease	1069 (14.9)	876 (14.1)	48 (19.8)	137 (21.1)	8 (25.8)
		Diabetes	844 (11.8)	676 (10.9)	41 (16.9)	116 (17.9)	11 (35.5)
		Cardiac condition	390 (5.5)	282 (4.5)	23 (9.5)	78 (12)	7 (22.6)
		Chronic kidney disease	302 (4.2)	225 (3.6)	19 (7.8)	52 (8)	6 (19.4)
		Peripheral vascular disease	230 (3.2)	178 (2.9)	12 (4.9)	38 (5.9)	2 (6.5)
		Chronic liver disease	132 (1.8)	116 (1.9)	8 (3.3)	8 (1.2)	-
		Cerebrovascular accident	137 (1.9)	101 (1.6)	9 (3.7)	24 (3.7)	3 (9.7)
		Drug use disorder	58 (0.8)	46 (0.7)	1 (0.4)	11 (1.7)	-
		HIV/AIDS	106 (1.5)	92 (1.5)	3 (1.2)	10 (1.5)	1 (3.2)
		Dementia—neurological condition	154 (2.2)	109 (1.7)	9 (3.7)	35 (5.4)	1 (3.2)
		Tuberculosis	63 (0.9)	47 (0.8)	2 (0.8)	13 (2)	1 (3.2)
		Transient ischemic attack	43 (0.6)	32 (0.5)	4 (1.6)	6 (0.9)	1 (3.2)
		Organ transplantation	34 (0.5)	24 (0.4)	-	9 (1.4)	1 (3.2)
		Sickle cell disease	4 (0.1)	3 (0)	1 (0.4)	-	-
		Down syndrome	4 (0.1)	4 (0.1)	-	-	-
Severe, n (%)			191,260 (100)	184,014 (96.2)	4071 (2.1)	3116 (1.6)	59 (0)
	Age, mean (SD)		49.6 (16.4)	49.0 (16.2)	62.2 (15.2)	66.1 (17.9)	72.3 (18)
	Female, n (%)		102,594 (53.6)	98,744 (53.7)	2116 (52)	1699 (54.5)	35 (59.3)
	Comorbidities, n (%)	Immunocompromised condition	61,460 (32.1)	58,577 (31.8)	1550 (38.1)	1305 (41.9)	28 (47.5)
		Hypertension	71,514 (37.4)	67,190 (36.5)	2367 (58.1)	1918 (61.6)	39 (66.1)
		Mental health conditions	36,318 (19)	34,553 (18.8)	924 (22.7)	822 (26.4)	19 (32.2)
		Cancer	29,403 (15.4)	27,958 (15.2)	776 (19.1)	654 (21)	15 (25.4)
		Obesity	34,683 (18.1)	33,577 (18.2)	688 (16.9)	410 (13.2)	8 (13.6)
		Chronic lung disease	29,106 (15.2)	27,581 (15)	738 (18.1)	759 (24.4)	28 (47.5)
		Diabetes	26,622 (13.9)	24,921 (13.5)	910 (22.4)	769 (24.7)	22 (37.3)
		Cardiac condition	9717 (5.1)	8790 (4.8)	426 (10.5)	489 (15.7)	12 (20.3)
		Chronic kidney disease	9250 (4.8)	8492 (4.6)	360 (8.8)	386 (12.4)	12 (20.3)
		Peripheral vascular disease	6134 (3.2)	5686 (3.1)	207 (5.1)	234 (7.5)	7 (11.9)
		Chronic liver disease	4282 (2.2)	4092 (2.2)	108 (2.7)	79 (2.5)	3 (5.1)
		Cerebrovascular accident	3341 (1.7)	3016 (1.6)	147 (3.6)	172 (5.5)	6 (10.2)
		Drug use disorder	1519 (0.8)	1450 (0.8)	35 (0.9)	33 (1.1)	1 (1.7)
		HIV/AIDS	2199 (1.1)	2139 (1.2)	37 (0.9)	23 (0.7)	-
		Dementia—neurological condition	2871 (1.5)	2467 (1.3)	151 (3.7)	244 (7.8)	9 (15.3)
		Tuberculosis	900 (0.5)	854 (0.5)	25 (0.6)	20 (0.6)	1 (1.7)
		Transient ischemic attack	1014 (0.5)	922 (0.5)	41 (1)	47 (1.5)	4 (6.8)
		Organ transplantation	786 (0.4)	740 (0.4)	19 (0.5)	26 (0.8)	1 (1.7)
		Sickle cell disease	95 (0)	90 (0)	4 (0.1)	1 (0)	-
		Down syndrome	35 (0)	33 (0)	1 (0)	-	1 (1.7)
Critical, n (%)			8841 (100)	8486 (96)	201 (2.3)	151 (1.7)	3 (0)
	Age, mean (SD)		58.8 (14.6)	58.3 (14.4)	67.7 (13.1)	73.9 (12.3)	77.3 (6.4)
	Female, n (%)		3109 (35.2)	2976 (35.1)	77 (38.3)	54 (35.8)	2 (66.7)
	Comorbidities, n (%)	Immunocompromised condition	2517 (28.5)	2360 (27.8)	71 (35.3)	85 (56.3)	1 (33.3)
		Hypertension	4330 (49)	4087 (48.2)	127 (63.2)	114 (75.5)	2 (66.7)
		Mental health condition	1576 (17.8)	1493 (17.6)	33 (16.4)	48 (31.8)	2 (66.7)
		Cancer	1027 (11.6)	947 (11.2)	35 (17.4)	44 (29.1)	1 (33.3)
		Obesity	1419 (16.1)	1375 (16.2)	29 (14.4)	15 (9.9)	(0)
		Chronic lung disease	2593 (29.3)	2468 (29.1)	64 (31.8)	60 (39.7)	1 (33.3)
		Diabetes	2167 (24.5)	2043 (24.1)	62 (30.8)	61 (40.4)	1 (33.3)
		Cardiac condition	622 (7)	569 (6.7)	20 (10)	33 (21.9)	-
		Chronic kidney disease	855 (9.7)	793 (9.3)	29 (14.4)	33 (21.9)	-
		Peripheral vascular disease	357 (4)	325 (3.8)	16 (8)	16 (10.6)	-
		Chronic liver disease	175 (2)	165 (1.9)	7 (3.5)	3 (2)	-
		Cerebrovascular accident	196 (2.2)	181 (2.1)	7 (3.5)	8 (5.3)	-
		Drug use disorder	54 (0.6)	51 (0.6)	2 (1)	1 (0.7)	-
		HIV/AIDS	62 (0.7)	60 (0.7)	-	2 (1.3)	-
		Dementia—neurological condition	103 (1.2)	90 (1.1)	5 (2.5)	8 (5.3)	-
		Tuberculosis	80 (0.9)	73 (0.9)	4 (2)	3 (2)	-
		Transient ischemic attack	41 (0.5)	39 (0.5)	1 (0.5)	1 (0.7)	-
		Organ transplantation	113 (1.3)	103 (1.2)	2 (1)	8 (5.3)	-
		Sickle cell disease	7 (0.1)	7 (0.1)	-	-	-
		Down syndrome	8 (0.1)	7 (0.1)	-	1 (0.7)	-

## Appendix B. Additional Data Focused on 2022

Proportion of high risk comorbidities	
Age group “18–29”	30.1%
Age group “30–49”	39.4%
Age group “50–64”	60.2%
Age group “65–74”	78.3%
Age group “75+”	90.8%
Probability infection being symptomatic (%) in 2022	
Age group “18–29”	3.1%
Age group “30–49”	4.9%
Age group “50–64”	3.9%
Age group “65–74”	4.3%
Age group “75+”	6.0%
Hospitalization rate among symptomatic patients (%) in 2022	
Age group “18–29”	0.3%
Age group “30–49”	0.6%
Age group “50–64”	1.6%
Age group “65–74”	3.5%
Age group “75+”	1.0%
Risk for hospitalization of with comorbidities patient group	2.6%
Risk for hospitalization of NON comorbidities patient group	0.5%
Critical care/ICU admission rate among hospitalized patients (%) in 2022	
Age group “18–29”	12.1%
Age group “30–49”	13.7%
Age group “50–64”	21.8%
Age group “65–74”	25.1%
Age group “75+”	15.1%
Risk for ICU of with comorbidities patient group	0.4%
Risk for ICU of with NON comorbidities patient group	0.1%
Probability of death among patients on normal ward (%) in 2022	
Age group “18–29”	1.2%
Age group “30–49”	1.1%
Age group “50–64”	5.7%
Age group “65–74”	12.0%
Age group “75+”	22.4%
Risk for death in GW of with comorbidities patient group	10.4%
Risk for death in GW of NON comorbidities patient group	4.6%
Probability of death among patients on critical care (%) in 2022	
Age group “18–29”	9.1%
Age group “30–49”	14.7%
Age group “50–64”	25.4%
Age group “65–74”	55.2%
Age group “75+”	52.1%
Risk for death in ICU of with comorbidities patient group	35.3%
Risk for death in ICU of NON comorbidities patient group	41.0%
Probability of death among patients on outpatient care (%) in 2022	
Age group “18–29”	0.0%
Age group “30–49”	0.0%
Age group “50–64”	0.2%
Age group “65–74”	0.7%
Age group “75+”	3.7%
Risk for death outpatient of with comorbidities patient group	0.5%
Risk for death outpatient of NON comorbidities patient group	0.0%
